# Human and digital human narrators in intangible cultural heritage short videos: dual psychological evaluation pathways through customer inspiration to electronic word-of-mouth intention

**DOI:** 10.3389/fpsyg.2026.1960625

**Published:** 2026-10-26

**Authors:** Huan Zhu, Wei Li, Zhihuang Huang, Bin Hu, Rongji Lin

**Affiliations:** 1 School of Communication and Design, Longyan University, Longyan, China; 2 School of Design Arts, Xiamen University of Technology, Xiamen, China

**Keywords:** customer inspiration, digital human narrator, electronic word-of-mouth intention, human narrator, intangible cultural heritage, short-form video

## Abstract

**Introduction:**

Explainer-style short-form videos have become an important medium for the digital communication of intangible cultural heritage (ICH), with narrators serving as key sources connecting ICH content to viewers’ psychological responses. However, how human and digital human narrators are associated with electronic word-of-mouth intention (eWOMI) through viewers’ perceptual, evaluative, and motivational processes remains insufficiently understood. Using explainer-style short-form videos about Hakka tulou as the research context, this study compared human and digital human narrators and examined two psychological evaluation pathways to customer inspiration (CI): one linking narrator nonverbal immediacy (NNI) to CI via narrator credibility (NC), and the other linking perceived narrator clarity (PNC) to CI through perceived information quality (PIQ). The study also investigated the relationship between CI and eWOMI.

**Methods:**

A randomized one-factor, two-level between-subjects experiment was conducted with a convenience sample of 601 participants. Data were analyzed using partial least squares structural equation modeling (PLS-SEM), bootstrapping, moderation analysis, and multigroup analysis.

**Results:**

Results indicated that both NNI and PNC were higher in the human narrator condition than in the digital human narrator condition. The relationships between NNI and NC and between NC and CI were stronger in the human narrator condition, whereas the relationships between PNC and PIQ and between PIQ and CI did not differ significantly between conditions. Focused attention positively moderated the relationships of NC and PIQ with CI. NNI and PNC were associated with CI through NC and PIQ, respectively, and CI was positively associated with eWOMI.

**Discussion:**

The findings suggest that the advantage of human narrators is concentrated in the source-evaluation pathway rather than the information-evaluation pathway, highlighting the roles of narrator-related perceptual cues and focused attention in shaping customer inspiration and dissemination intention in ICH short-form videos.

## 1 Introduction

Amid the accelerating global digital transformation and increasing emphasis on cultural heritage preservation, the role of digital media in shaping public perceptions, evaluations, and dissemination intentions regarding intangible cultural heritage (ICH) has become a critical focus in cultural communication research. With the emergence of short-form video platforms, ICH communication is no longer limited to museum exhibitions, documentary narratives, or written descriptions. Characterized by easy accessibility, strong audiovisual appeal, and high dissemination potential, short-form videos offer a novel avenue for ICH content to transcend established cultural-interest communities and reach wider audiences (Paquienseguy and Guo, 2025). In explainer-style ICH short-form videos, narrators act as key communicators who connect ICH knowledge and cultural narratives to viewers’ psychological engagement. The narrators analyzed in this study are neither entertainment livestreamers nor conventional information presenters; rather, they serve as communication agents responsible for elucidating cultural knowledge, situating it within its historical context, and interpreting its cultural significance (He et al., 2026).

Narrators’ facial expressions, gaze, gestures, vocal rhythm, and verbal delivery influence not only viewers’ perceptions of their approachability, naturalness, and credibility (Santilli et al., 2011) but also viewers’ assessments of the clarity, accuracy, and explanatory depth of ICH content (Chesebro and McCroskey, 2001). Thus, narrators’ nonverbal and verbal expressions serve as more than mere modes of content presentation; they function as critical media cues that shape viewers’ psychological evaluations of both the narrator and the information conveyed. With advances in digital human technologies, digital humans have increasingly been employed in museum tours, cultural heritage exhibitions, and short-form video production, where they perform communicative functions such as explaining knowledge, presenting cultural content, and interacting with users (Sylaiou and Fidas, 2022; Wang, L. et al., 2025). As an emerging type of communication source, digital human narrators may differ from human narrators in their visual presentation, nonverbal behavior, and verbal delivery, thereby providing distinct source cues that influence viewers’ evaluations of both the communicator and the information presented. Consequently, the differences between human and digital human communicators, along with their psychological effects, have become a significant topic in digital communication research (Zeng and Lin, 2025; Zhang et al., 2025).

Previous research on human and digital human communicators has drawn on perspectives such as source credibility theory (Hovland and Weiss, 1951), social presence theory (Short et al., 1976; Gunawardena, 1995), and the Computers Are Social Actors (CASA) paradigm (Nass and Moon, 2000) to explain audience responses toward mediated communicators. However, existing studies have mainly focused on overall perceptions, while paying less attention to how narrator cues shape distinct evaluations of the communicator and the conveyed information. Other studies have examined the dissemination of intangible cultural heritage (ICH) content on platforms such as Douyin in mainland China by analyzing configurations of platform characteristics, content features, and user engagement (Wang, S. et al., 2025), or have focused on the technological acceptance, anthropomorphism, and perceived trustworthiness of digital humans. Although previous studies have examined human and digital human communicators in terms of trust, acceptance, anthropomorphism, and social presence (Sundar, 2020; Liew and Tan, 2021; Miao et al., 2022), they have primarily focused on overall perceptions rather than on how specific narrator cues shape evaluations of both the communicator and the conveyed information. Drawing on source credibility theory (Hovland and Weiss, 1951), communication effects involve both the source and the message. This distinction is particularly relevant in ICH short-form videos, where narrators serve as both information providers and interpreters of cultural meaning. Therefore, distinguishing between source evaluation and information evaluation is essential to explain how narrator type influences dissemination intentions.

Using explainer-style short-form videos on intangible cultural heritage (ICH) featuring Hakka tulou as the research context, this study experimentally manipulates narrator type and develops and tests a dual-route psychological evaluation model. The model differentiates between two viewer-perceived narrator cues: narrator nonverbal immediacy (NNI) and perceived narrator clarity (PNC). It examines whether NNI is associated with customer inspiration through narrator credibility, whether PNC is associated with customer inspiration through perceived information quality, and whether customer inspiration is associated with electronic word-of-mouth intention. The study also examines whether focused attention moderates the relationships between narrator credibility and customer inspiration, and between perceived information quality and customer inspiration. Additionally, it compares key structural paths across human and digital human narrator conditions. By integrating narrator presentation, viewer perceptions, psychological evaluations, and dissemination intentions, this study provides a process-based explanation of how human and digital human narrators shape viewers’ psychological responses and electronic word-of-mouth intention in ICH short-form videos. It also offers practical guidance for designing ICH short-form videos and the appropriate use of digital human narrators.

## 2 Theoretical background and hypotheses

Building on the distinction between source-related and information-related evaluations in communication processes, this study proposes a dual-route psychological evaluation framework to explain how narrator cues are associated with viewers’ responses in ICH short-form videos. The source-related evaluation route is grounded in source credibility theory (Hovland and Weiss, 1951), suggesting that audiences evaluate the communicator based on perceived credibility. Accordingly, narrator nonverbal immediacy is positively related to narrator credibility, which is, in turn, positively related to customer inspiration. The information-related evaluation route draws on information adoption theory (Sussman and Siegal, 2003), suggesting that audiences evaluate the quality of conveyed information before forming responses. Accordingly, perceived narrator clarity is positively related to perceived information quality, which is, in turn, positively related to customer inspiration. Customer inspiration connects these two evaluation routes with electronic word-of-mouth intention.

### 2.1 Narrator type and perceived narrator cues

Research on source cues suggests that when evaluating mediated information, audiences not only process the message content but also form judgments based on source cues such as the communicator’s identity, credibility, and mode of presentation (Chaiken, 1980; Hovland and Weiss, 1951). With advances in digital communication technologies, technologically generated communicators—including virtual streamers, digital humans, and virtual influencers—have increasingly assumed the role of information sources (Sangeeth Chandran et al., 2026; Zeng and Lin, 2025). This study defines narrator type as two categories of communication sources responsible for explaining ICH: human narrators and digital human narrators. In explainer-style ICH short-form videos, narrators not only explain ICH knowledge and interpret its cultural significance; their human or digital human identity, together with their bodily presentation, gestures, facial expressions, vocal delivery, and overall audiovisual presentation, also provide important source cues through which viewers evaluate both the narrator and the information conveyed. The influence of narrator type may therefore extend beyond differences in identity and technological presentation and be reflected in two perceived narrator cues: narrator nonverbal immediacy and perceived narrator clarity.

Building on these differences in source cues, and to avoid weakening the explanatory power of the proposed mechanisms by conflating distinct perceptual cues, this study distinguishes between narrator nonverbal immediacy and perceived narrator clarity (Chesebro and McCroskey, 1998; Richmond et al., 2003). narrator nonverbal immediacy (NNI) refers to viewers’ perceptions of the narrator’s facial expressions, movements, posture, gaze, bodily rhythm, and sense of interpersonal immediacy while watching ICH short-form videos. Perceived narrator clarity (PNC) refers to viewers’ perceptions of whether the narrator’s verbal delivery is clear, the explanation is well organized, the content is easy to understand, and technical terms are accurately explained. Differences between human and digital human narrators in bodily presentation, naturalness of movement, facial expressiveness, and presentation realism may produce different levels of NNI (Richmond et al., 2003; Sangeeth Chandran et al., 2026). Differences in vocal naturalness, delivery structure, and the manner in which technical terms are explained may likewise shape viewers’ PNC evaluations (Chesebro and McCroskey, 2001; Ehret et al., 2021). Because no *a priori* assumption is made that either narrator type is superior, the following nondirectional hypotheses are proposed:

*H1a*: Narrator nonverbal immediacy differs between the human and digital human narrator conditions.

*H1b*: Perceived narrator clarity differs between the human and digital human narrator conditions.

### 2.2 Narrator nonverbal immediacy, narrator credibility, and customer inspiration

Narrator nonverbal immediacy reflects viewers’ psychological evaluation of a narrator’s nonverbal cues, while narrator credibility is primarily explained by source credibility theory. This theory posits that the credibility of an information source influences audiences’ acceptance of the message and their attitudinal responses (Hovland and Weiss, 1951). Ohanian (1990) further operationalized source credibility along three dimensions: expertise, trustworthiness, and attractiveness. In the present context, narrator credibility refers to viewers’ perceptions of whether a narrator is professional, credible, reliable, and well-suited to the role of interpreting cultural knowledge in explainer-style ICH short-form videos. Previous research indicates that nonverbal immediacy can reduce psychological distance between communicators and audiences and shape audiences’ evaluations of communicator credibility (Richmond et al., 2003; Santilli et al., 2011). Accordingly, when viewers perceive a narrator as exhibiting greater nonverbal immediacy, they are more likely to regard the narrator as natural, approachable, and credible. Therefore, the following hypothesis is proposed:

*H2a*: Narrator nonverbal immediacy is positively related to narrator credibility.

Böttger et al. (2017) defined customer inspiration as a transient motivational state triggered by an external stimulus. It consists of two sequentially related but conceptually distinct states: inspired-by and inspired-to. In the context of explainer-style ICH short-form videos, customer inspiration refers to viewers’ feelings of being inspired by the ICH content, its mode of narration, and its cultural storytelling, along with their motivation to learn more about, experience, or otherwise engage with the related cultural content. According to source credibility theory, credible narrators increase the likelihood that viewers will accept their interpretations of ICH knowledge and cultural significance (Hovland and Weiss, 1951) and perceive the narrated content as an inspiring external stimulus. Previous research has similarly identified source credibility as an important antecedent of customer inspiration (Nguyen et al., 2025; Song et al., 2024; Yang et al., 2024). Accordingly, the following hypothesis is proposed:

*H2b*: Narrator credibility is positively related to customer inspiration.

When viewers perceive a narrator as exhibiting greater nonverbal immediacy, they are more likely to experience a sense of approachability, naturalness, and interpersonal presence, thereby enhancing their perceptions of the narrator’s credibility (Santilli et al., 2011). Increased narrator credibility, in turn, encourages viewers to accept the narrator’s interpretation of ICH and to regard the narrated content as an inspiring external stimulus (Song et al., 2024; Yang et al., 2024). Therefore, narrator nonverbal immediacy may be related to customer inspiration through narrator credibility, suggesting that narrator credibility mediates the relationship between these two constructs. Accordingly, the following hypothesis is proposed:

*H2c*: Narrator credibility mediates the relationship between narrator nonverbal immediacy and customer inspiration.

### 2.3 Perceived narrator clarity, perceived information quality, and customer inspiration

Perceived narrator clarity was defined in the preceding section. Research on information adoption suggests that information quality influences individuals’ judgments of information usefulness and adoption value (Sussman and Siegal, 2003) and is commonly reflected in attributes such as accuracy, completeness, relevance, and understandability (DeLone and McLean, 1992; DeLone and McLean, 2003). In this study, perceived information quality refers to viewers’ evaluations of whether the content of an ICH short-form video is accurate, complete, relevant, valuable, easy to understand, and effective in conveying cultural knowledge. While perceived narrator clarity concerns the communication process, perceived information quality concerns viewers’ evaluation of the conveyed content, including its accuracy, relevance, and usefulness. Thus, perceived narrator clarity reflects how effectively information is delivered, whereas perceived information quality reflects how valuable and credible the information itself is perceived to be. Clear delivery can reduce the audience’s information-processing burden and facilitate content comprehension and cognitive learning (Chesebro and McCroskey, 1998; Chesebro and McCroskey, 2001). Therefore, when a narrator communicates clearly, provides straightforward explanations, and emphasizes key points, viewers can more readily understand the core elements of ICH knowledge and are more likely to perceive the video content as high-quality information. Accordingly, the following hypothesis is proposed:

*H3a*: Perceived narrator clarity is positively related to perceived information quality.

Previous research on customer inspiration in short-form video contexts has demonstrated that content that is information-rich, reliable, vivid, and fluently presented is more likely to foster customer inspiration (Gao et al., 2021). The accuracy, reliability, understandability, and completeness associated with perceived information quality (DeLone and McLean, 1992) enable viewers to perceive ICH short-form video content as a valuable and inspiring external stimulus, thereby facilitating the development of customer inspiration. Accordingly, the following hypothesis is proposed:

*H3b*: Perceived information quality is positively related to customer inspiration.

Perceived narrator clarity can reduce the cognitive burden associated with understanding ICH knowledge and help viewers identify the content’s core information and logical structure, thereby enhancing perceived information quality (Chesebro and McCroskey, 1998; Chesebro and McCroskey, 2001). Higher perceived information quality, in turn, encourages viewers to perceive the content as an external stimulus with both informational value and inspirational potential, thereby stimulating customer inspiration (Gao et al., 2021). Consequently, perceived information quality may mediate the relationship between perceived narrator clarity and customer inspiration. Accordingly, the following hypothesis is proposed:

*H3c*: Perceived information quality mediates the relationship between perceived narrator clarity and customer inspiration.

### 2.4 Customer inspiration and electronic word-of-mouth intention

Electronic word of mouth (eWOM) refers to individuals sharing information, evaluations, and experiences about products, services, or specific content with others via the internet (Berger and Milkman, 2012; Hennig-Thurau et al., 2004). Electronic word-of-mouth intention (eWOMI) reflects an individual’s behavioral inclination to recommend, share, or discuss relevant content through digital platforms in the future (Chu and Kim, 2011; Hennig-Thurau et al., 2004). In the context of explainer-style ICH short-form videos, eWOMI is defined as viewers’ willingness, after watching an ICH short-form video, to recommend, share, positively evaluate, or discuss the content with others through social media, group chats, or other online networks (Hennig-Thurau et al., 2004; Muntinga et al., 2011). Because the measurement items in this study primarily concern recommendation, positive evaluation, sharing, and favorable discussion, the study specifically examines positive eWOM while consistently referring to the construct as electronic word-of-mouth intention.

Customer inspiration encompasses not only the experience of being inspired by an external stimulus but also the motivation to translate that experience into action (Böttger et al., 2017). Previous studies have demonstrated that customer inspiration can promote positive behavioral responses, including engagement, recommendation, and word-of-mouth communication (Böttger et al., 2017; Izogo and Mpinganjira, 2020). In social media contexts, inspiring content is more likely to stimulate users’ motivation to share and facilitate content dissemination (Hennig-Thurau et al., 2004; Oltra González et al., 2022). In the context of ICH short-form videos, viewers inspired by the content are more likely to recognize its cultural value and perceive it as worth communicating to others, thereby developing stronger intentions to recommend, share, and discuss it. Accordingly, the following hypothesis is proposed:

*H4*: Customer inspiration is positively related to electronic word-of-mouth intention.

### 2.5 The moderating role of focused attention

The theoretical foundation of focused attention is primarily derived from Kahneman’s capacity theory of attention, which posits that individuals have limited attentional resources for information processing and that the extent of attentional investment influences how information is received, understood, and remembered (Kahneman, 1973). In short-form video environments characterized by rapid browsing and frequent content switching, viewers’ sustained attention is particularly susceptible to disruption (Chiossi et al., 2023). Accordingly, this study defines focused attention as the subjective degree to which viewers maintain their attention on cultural content, narrator presentation, and video narrative cues while watching ICH short-form videos (Lin and Chen, 2026).

Although narrator credibility and perceived information quality reflect viewers’ favorable evaluations of the narrator and the content, respectively, transforming these evaluations into customer inspiration requires further information processing and meaning integration. Therefore, focused attention may influence the strength of both evaluative pathways. Regarding the perceived information quality pathway, social media research suggests that user involvement and immersive states affect the processing of content cues and their subsequent effects (Javed et al., 2024; Zha et al., 2018). When focused attention is high, viewers are more likely to develop a deeper understanding of ICH knowledge and integrate its cultural significance, making perceived information quality more likely to translate into customer inspiration. Previous research has also found that user involvement strengthens the effect of source credibility on perceived information credibility (Javed et al., 2024). When focused attention is high, viewers may process cultural interpretations provided by a credible narrator more deeply and regard them as inspiring external stimuli. Accordingly, the following hypotheses are proposed:

*H5a*: Focused attention positively moderates the relationship between perceived information quality and customer inspiration, with a stronger relationship at higher levels of focused attention.

*H5b*: Focused attention positively moderates the relationship between narrator credibility and customer inspiration, with a stronger relationship at higher levels of focused attention.

### 2.6 Multigroup differences in structural paths by narrator type

Human and digital human narrators represent distinct types of media sources. Differences in their authenticity, naturalness of expression, and modes of media presentation may lead viewers to interpret their verbal and nonverbal cues differently (Sangeeth Chandran et al., 2026; Zeng and Lin, 2025). For human narrators, perceived nonverbal immediacy is often interpreted primarily as an expression of authentic interaction and interpersonal approachability (Santilli et al., 2011). However, similar behaviors displayed by digital human narrators may be evaluated more in terms of behavioral naturalness and representational fidelity (Oh Kruzic et al., 2020). These differences in attribution may produce between-group variation in the effect of narrator nonverbal immediacy on narrator credibility. Moreover, because credible and approachable communicators are more likely to inspire viewers (Ki et al., 2022), the effect of narrator credibility on customer inspiration may also vary by narrator type.

For human narrators, perceived narrator clarity is often influenced more strongly by natural storytelling, variations in intonation, and the comprehensibility of cultural explanations (Weiler and Walker, 2014). For digital human narrators, it may be more closely related to the prosodic configuration of synthetic speech, particularly the appropriate placement of focal stress, which can affect perceived naturalness (Ehret et al., 2021). These differences may modify the impact of perceived narrator clarity on perceived information quality. Previous research has also demonstrated that information-rich, reliable, vivid, and narrative-driven content is more likely to stimulate customer inspiration (Gao et al., 2021). Therefore, it can be inferred that narrator type may influence the degree to which high-quality information inspires viewers, leading to between-group differences in the effect of perceived information quality on customer inspiration. Accordingly, the following hypotheses are proposed:

*H6a*: The relationship between narrator nonverbal immediacy and narrator credibility differs between the human and digital human narrator conditions.

*H6b*: The relationship between narrator credibility and customer inspiration differs between the human and digital human narrator conditions.

*H6c*: The relationship between perceived narrator clarity and perceived information quality differs between the human and digital human narrator conditions.

*H6d*: The relationship between perceived information quality and customer inspiration differs between the human and digital human narrator conditions.

Based on the preceding theoretical arguments and hypotheses, the theoretical model developed in this study is illustrated in Figure 1.

**Figure 1:**
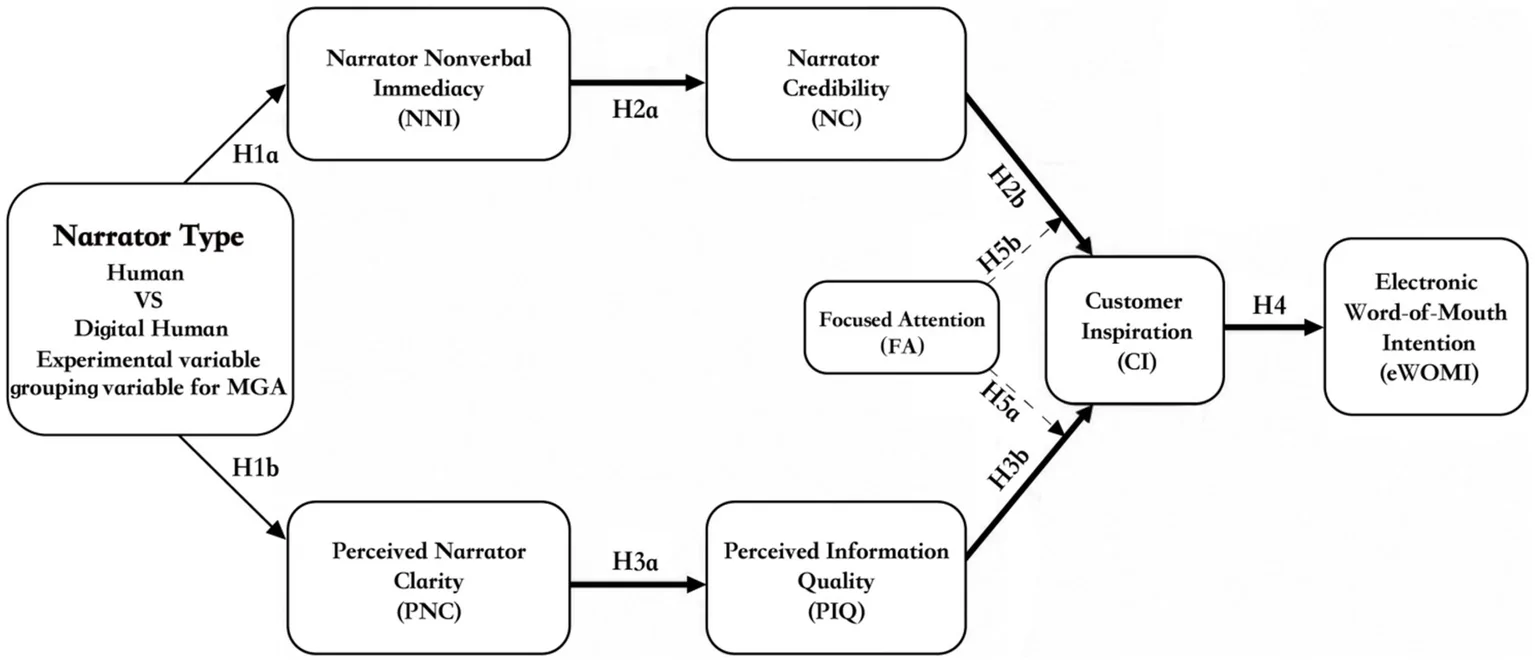
Conceptual framework. Narrator type was experimentally manipulated and used for MGA grouping. H1a–H1b, group differences; solid structural arrows, direct effects; dashed arrows, moderation; H2c/H3c, indirect effects; H6a–H6d, MGA.

## 3 Materials and methods

### 3.1 Experimental design and stimuli

This study employed a randomized, one-factor, two-level between-subjects experimental design. Participants were randomly assigned to one of two conditions and viewed the corresponding version of an explainer-style ICH short-form video. The experimental stimuli consisted of two videos featuring Hakka tulou culture (Figure 2). The human narrator and digital human narrator versions were 60.674 and 60.906 s long, respectively. Both videos had a resolution of 3,840 × 2,160 pixels, a frame rate of 30 frames per second, and the same 252-character Chinese narration script. Their average narration rates were 249.2 and 248.3 Chinese characters per minute, respectively. The subtitles, background footage, background music, editing pace, and narrative structure were held constant across the two versions, and the audio volume was matched. Although no separate formal stimulus pretest was conducted, stimulus comparability was ensured through these controlled production procedures. The two videos represented complete narrator presentation conditions rather than isolated manipulations of narrator identity. Because only one human narrator and one digital human narrator were used, the findings reflect differences between the two presentation conditions, which also involved related audiovisual cues, including facial appearance, voice, gestures, intonation, and animation quality. The digital human narrator was created using an AI-assisted workflow. GPT was used to analyze the original video and generate prompts for digital human video creation. Seedance 2.0 generated the digital human video, while Suno AI reconstructed the voice and Adobe Premiere Pro completed final editing. The narrator’s facial expressions, posture, gestures, bodily movements, and vocal characteristics were considered cues for narrator nonverbal immediacy, while verbal delivery, speech rate, and content organization formed the basis for evaluations of perceived narrator clarity. After watching the video, participants completed a questionnaire measuring narrator nonverbal immediacy, perceived narrator clarity, narrator credibility, perceived information quality, customer inspiration, focused attention, and electronic word-of-mouth intention.

**Figure 2:**
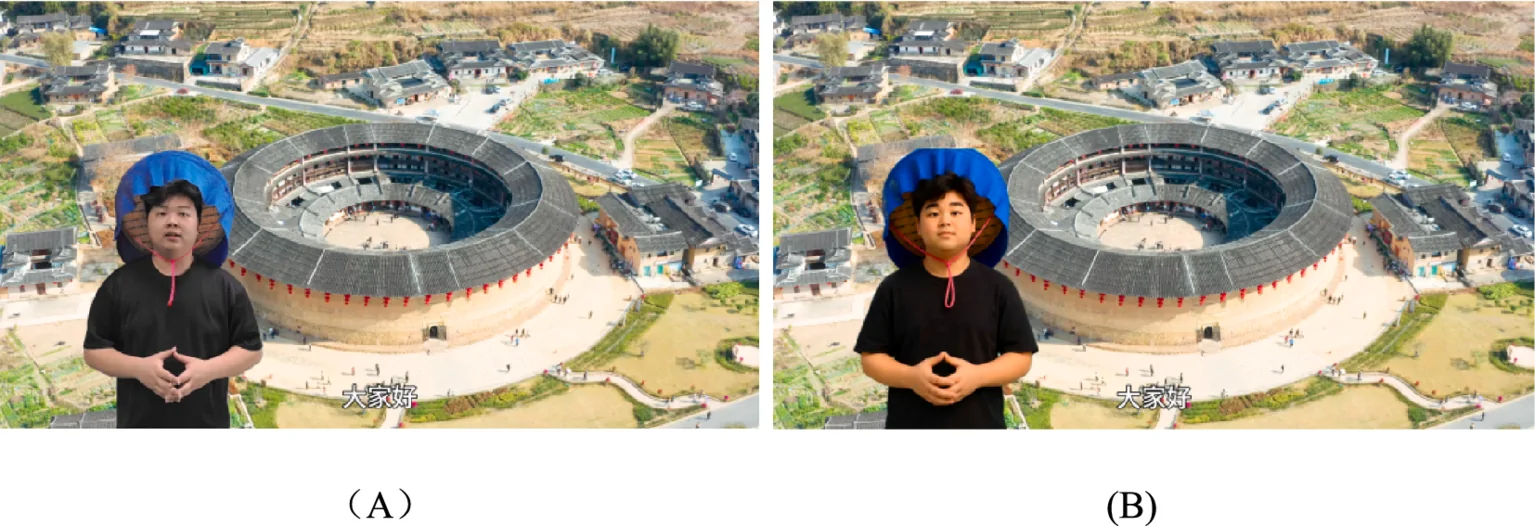
Representative frames from the experimental stimuli: **(A)** human narrator condition; **(B)** digital human narrator condition.

### 3.2 Participants and procedure

Participants were recruited online in mainland China through convenience sampling between June 4 and June 8, 2026. The questionnaire was distributed online via WeChat, using Wenjuanxing to embed the videos, collect responses, and screen potential duplicate submissions. Participants watched the assigned video and completed the questionnaire using a smartphone. All principal constructs were measured using five-point Likert scales. Eligible participants were required to reside in mainland China, understand Chinese, use a smartphone, and participate voluntarily. Before the experiment, all participants read the study information and provided informed consent. Each participant received CNY 0.30 upon completion. A total of 603 questionnaires were collected. Two responses were excluded because participants failed the attention-check item (Q8), resulting in a final sample of 601 participants. An *a priori* power analysis using G*Power 3.1 indicated a minimum required sample size of 92 participants; the final sample of 601 participants was sufficient for the proposed analyses. Participants were randomly assigned to the human narrator (*n* = 300) or digital human narrator (*n* = 301) condition. The final sample comprised 314 men (52.2%) and 287 women (47.8%), aged 25–38 years (*M* = 31.29), which may reflect the characteristics of the online recruitment sample. No significant differences were found between conditions in demographic or baseline variables (all ps > 0.05), indicating comparable groups after randomization.

The experimental procedure included obtaining informed consent, collecting pre-viewing baseline measures, randomized video exposure, post-viewing assessments, and gathering information on short-form video usage habits and demographics (Figure 3). Participants first read the study information and provided informed consent. Responses were collected anonymously and used solely for academic research and statistical analysis. Participants then reported their familiarity with and interest in ICH, traditional culture, and museum- and cultural heritage-related content. The survey system subsequently assigned participants at random to either the human narrator or digital human narrator condition. Participants were required to watch the assigned video in its entirety before proceeding to the next page. After viewing the video, participants identified the narrator type they perceived and completed measures assessing narrator nonverbal immediacy, perceived narrator clarity, narrator credibility, perceived information quality, customer inspiration, electronic word-of-mouth intention, and focused attention (Supplementary Table S1). The average completion time was approximately 6 min. Narrator identification was assessed as a manipulation check, with identification rates of 53.33 and 53.49% in the human and digital human conditions, respectively. Subsequent analyses were based on assigned conditions. The questionnaire also included an attention-check item for subsequent data screening. Finally, participants reported their short-form video usage habits and demographic information. These questions were placed at the end of the questionnaire to minimize their potential influence on participants’ immediate post-viewing psychological responses.

**Figure 3:**
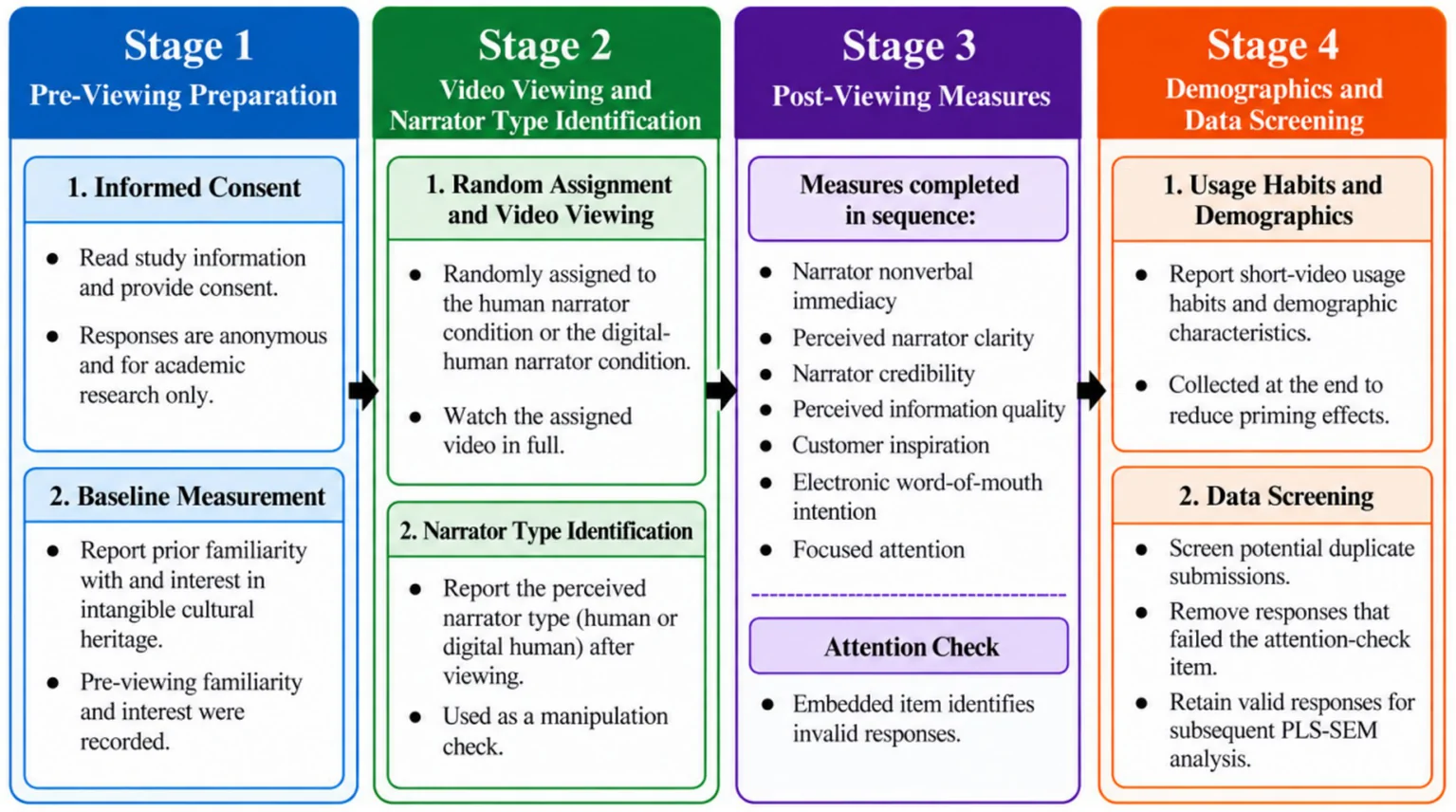
Experimental procedure.

### 3.3 Measures

The measurement items for the core constructs were adapted from established scales to fit the context of ICH short-form video communication. Some items were directly adopted, while others were modified or selected for contextual relevance to ICH short-form video communication. The questionnaire was translated and back-translated by bilingual researchers, and a pilot test was conducted before the formal survey. Detailed measurement items and adaptation procedures are provided in Supplementary Table S1. Except for demographic information and single-item background variables, all core constructs were measured using five-point Likert scales ranging from 1 (strongly disagree) to 5 (strongly agree). The definitions of the constructs and their primary measurement sources are presented in Table 1.

**Table 1:** Definitions and sources of constructs.

Construct	Definition	Reference
Narrator Nonverbal Immediacy (NNI)	The extent to which viewers perceive the narrator’s facial expressions, eye contact, gestures, posture, body movement, and nonverbal behavior as natural and immediate.	Richmond et al. (2003)
Perceived Narrator Clarity (PNC)	Viewers’ perceptions of whether the narrator’s explanation is clear, well organized, easy to understand, and accurate.	Chesebro and McCroskey (1998)
Narrator Credibility (NC)	Viewers’ perceptions of the narrator’s professionalism, credibility, reliability, and suitability for interpreting cultural knowledge.	Ohanian (1990)
Perceived Information Quality (PIQ)	The extent to which viewers perceive the video content as accurate, complete, relevant, valuable, informative, and helpful for understanding intangible cultural heritage.	Cheung et al. (2008)
Customer Inspiration (CI)	The extent to which viewers feel inspired by the content and develop interest, new ideas, or motivation to further explore intangible cultural heritage.	Böttger et al. (2017)
Electronic Word-of-Mouth Intention (eWOMI)	Viewers’ willingness to recommend, share, positively evaluate, or discuss intangible cultural heritage short videos through online networks.	Goyette et al. (2010)
Focused Attention (FA)	Viewers’ perceived degree of sustained attentional engagement with intangible cultural heritage content, narrator expression, and video narrative cues while watching intangible cultural heritage short videos.	O’Brien et al. (2018)

### 3.4 Data analysis

SmartPLS 4.1.1.4 was used for PLS-SEM analysis, which was selected to examine mediation, moderation, between-group differences, and the explanatory and predictive performance of key endogenous constructs (Hair et al., 2019). The measurement model was assessed for reliability and validity, followed by independent-samples *t*-tests comparing the two narrator conditions. The structural model was evaluated using 5,000 bootstrap subsamples, with two-tailed tests at *α* = 0.05 and 95% percentile bootstrap confidence intervals; effects were considered significant when *p* < 0.05 and the confidence interval excluded zero. Moderation was tested using the two-stage approach. Path coefficients, *R*^2^, and *f*^2^ were reported, and predictive performance was assessed using PLSpredict with 10-fold cross-validation and 10 repetitions, including *Q*^2^_predict and prediction-error comparisons with a linear model benchmark. MICOM was conducted prior to the multigroup analysis.

## 4 Results

### 4.1 Measurement model assessment

The measurement model was initially evaluated based on indicator reliability, internal consistency reliability, and convergent validity. Most indicators exhibited outer loadings above 0.70. Although a few indicators had loadings below 0.70, their values remained within an acceptable range. Specifically, NC5 (0.699), PIQ5 (0.665), and CI6 (0.677) showed outer loadings below 0.70. These indicators were retained because the composite reliability and average variance extracted (AVE) values of the corresponding constructs met the recommended criteria (Hair et al., 2019). The outer loadings of all indicators are reported in Supplementary Table S2.

As shown in Table 2, Cronbach’s *α* values ranged from 0.765 to 0.821, rho_A values ranged from 0.772 to 0.826, composite reliability values ranged from 0.848 to 0.870, and AVE values ranged from 0.527 to 0.583. All values met the recommended thresholds. Table 3 shows that all heterotrait–monotrait ratio (HTMT) values were below 0.80, indicating satisfactory discriminant validity for the measurement model (Henseler et al., 2015). Bootstrapped HTMT confidence intervals further supported discriminant validity, as none of the 95% confidence intervals included 1 (Supplementary Table S4). Notably, the HTMT between perceived narrator clarity (PNC) and perceived information quality (PIQ) was 0.326 (95% CI [0.233, 0.421]). The MICOM procedure further established configural and compositional invariance for all constructs, supporting partial measurement invariance, although equality of composite means and variances was not achieved for all constructs (Supplementary Table S3). The cross-loadings of all indicators are reported in Supplementary Table S2.

**Table 2:** Reliability and convergent validity assessment.

Construct	Cronbach’s alpha	rho_A	rho_C	AVE
Perceived information quality	0.793	0.797	0.858	0.548
Electronic word-of-mouth intention	0.791	0.793	0.857	0.545
Focused attention	0.765	0.772	0.848	0.583
Narrator credibility	0.793	0.793	0.858	0.546
Perceived narrator clarity	0.812	0.814	0.869	0.571
Narrator nonverbal immediacy	0.804	0.810	0.863	0.558
Customer inspiration	0.821	0.826	0.870	0.527

**Table 3:** Heterotrait–Monotrait ratio (HTMT).

Construct	PIQ	eWOMI	FA	NC	PNC	NNI	CI
PIQ							
eWOMI	0.312						
FA	0.357	0.46					
NC	0.297	0.385	0.311				
PNC	0.326	0.317	0.261	0.395			
NNI	0.313	0.381	0.317	0.304	0.367		
CI	0.323	0.429	0.358	0.344	0.42	0.426	

### 4.2 Group differences in perceived narrator cues

After establishing satisfactory reliability and validity for the measurement model, independent-samples *t*-tests were conducted to compare narrator nonverbal immediacy (NNI) and perceived narrator clarity (PNC) across the two narrator conditions. Levene’s tests indicated unequal variances for both NNI and PNC; therefore, Welch’s *t*-tests were used. As shown in Table 4, NNI was significantly higher in the human narrator condition (*M* = 3.745) than in the digital human narrator condition (*M* = 3.397), *t* = 5.806, *p* < 0.001, mean difference = 0.348, 95% CI [0.230, 0.466], and Cohen’s *d* = 0.474, supporting H1a. Similarly, PNC was significantly higher in the human narrator condition (*M* = 3.725) than in the digital human narrator condition (*M* = 3.355), *t* = 6.085, *p* < 0.001, mean difference = 0.370, 95% CI [0.251, 0.489], and Cohen’s *d* = 0.497, supporting H1b. These results indicate that, within the specific ICH short-form video stimuli and narrator conditions examined in this study, participants reported higher levels of NNI and PNC for the human narrator than for the digital human narrator.

**Table 4:** Group differences in perceived narrator cues.

Construct	Human *M* (SD)	Digital *M* (SD)	Mean difference [95% CI]	*t*	*p*	Cohen’s *d*	Levene’s *F* (*p*)
NNI	3.745 (0.702)	3.397 (0.766)	0.348 [0.230, 0.466]	5.806	<0.001	0.474	6.929 (0.009)
PNC	3.725 (0.696)	3.355 (0.791)	0.370 [0.251, 0.489]	6.085	<0.001	0.497	9.781 (0.002)

### 4.3 Structural model and hypothesis testing

Before examining the structural paths, collinearity within the structural model was assessed. The inner variance inflation factor (VIF) values for all predictor constructs ranged from 1.000 to 1.187, well below the recommended threshold, indicating no significant collinearity concerns. The structural model results are presented in Figure 4, while the direct and moderating effects are reported in Table 5. The results showed positive relationships between NNI and NC (*β* = 0.249, *t* = 6.794, *p* < 0.001), PNC and PIQ (*β* = 0.264, *t* = 7.007, *p* < 0.001), NC and CI (*β* = 0.174, *t* = 4.670, *p* < 0.001), PIQ and CI (*β* = 0.154, *t* = 3.644, *p* < 0.001), and CI and eWOMI (*β* = 0.358, *t* = 10.696, *p* < 0.001). Therefore, H2a, H2b, H3a, H3b, and H4 were supported.

**Figure 4:**
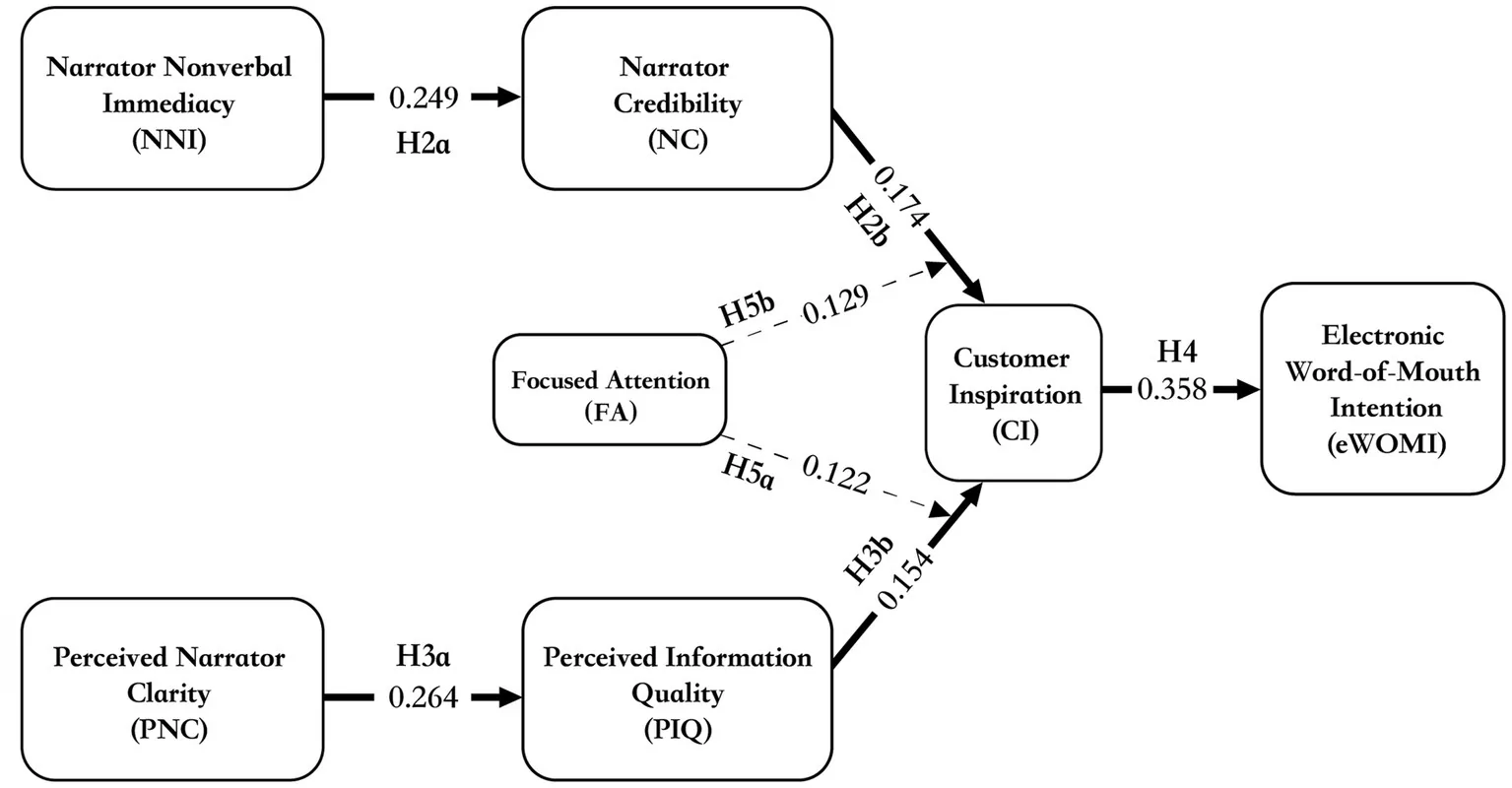
Results of the PLS-SEM analysis.

**Table 5:** Structural path coefficients, effect sizes, and moderation effects.

Hypothesis	Path	β	M	SD	t-value	*p*-value	*f* ^2^	Result
H2a	NNI → NC	0.249	0.258	0.037	6.794	<0.001	0.066	Supported
H2b	NC → CI	0.174	0.176	0.037	4.670	<0.001	0.032	Supported
H3a	PNC → PIQ	0.264	0.272	0.038	7.007	<0.001	0.075	Supported
H3b	PIQ → CI	0.154	0.155	0.042	3.644	<0.001	0.025	Supported
H4	CI → eWOMI	0.358	0.363	0.033	10.696	<0.001	0.147	Supported
–	FA → CI	0.242	0.245	0.039	6.157	<0.001	0.062	–
H5a	FA × PIQ → CI	0.122	0.122	0.036	3.359	0.001	0.019	Supported
H5b	FA × NC → CI	0.129	0.126	0.038	3.384	0.001	0.016	Supported

As part of the moderation model, FA was positively related to CI (*β* = 0.242, *t* = 6.157, *p* < 0.001). The interaction between FA and PIQ also had a significant positive effect on CI (*β* = 0.122, *t* = 3.359, *p* = 0.001), as did the interaction between FA and NC (*β* = 0.129, *t* = 3.384, *p* = 0.001). Therefore, both H5a and H5b were supported. As shown in Figure 5, the positive relationships of NC and PIQ with CI became steeper as FA increased from −1 SD to +1 SD. The effect sizes (*f*^2^) of the structural paths were also examined. Most paths showed small effects, whereas the relationship between CI and eWOMI demonstrated the largest effect size among the examined paths. The *R*^2^ values for PIQ, NC, CI, and eWOMI were 0.070, 0.062, 0.192, and 0.128, respectively. The relatively low *R*^2^ values for PIQ and NC indicate that the proposed predictors explain only part of the variance in these constructs, reflecting limited explanatory power for these two endogenous variables.

**Figure 5:**
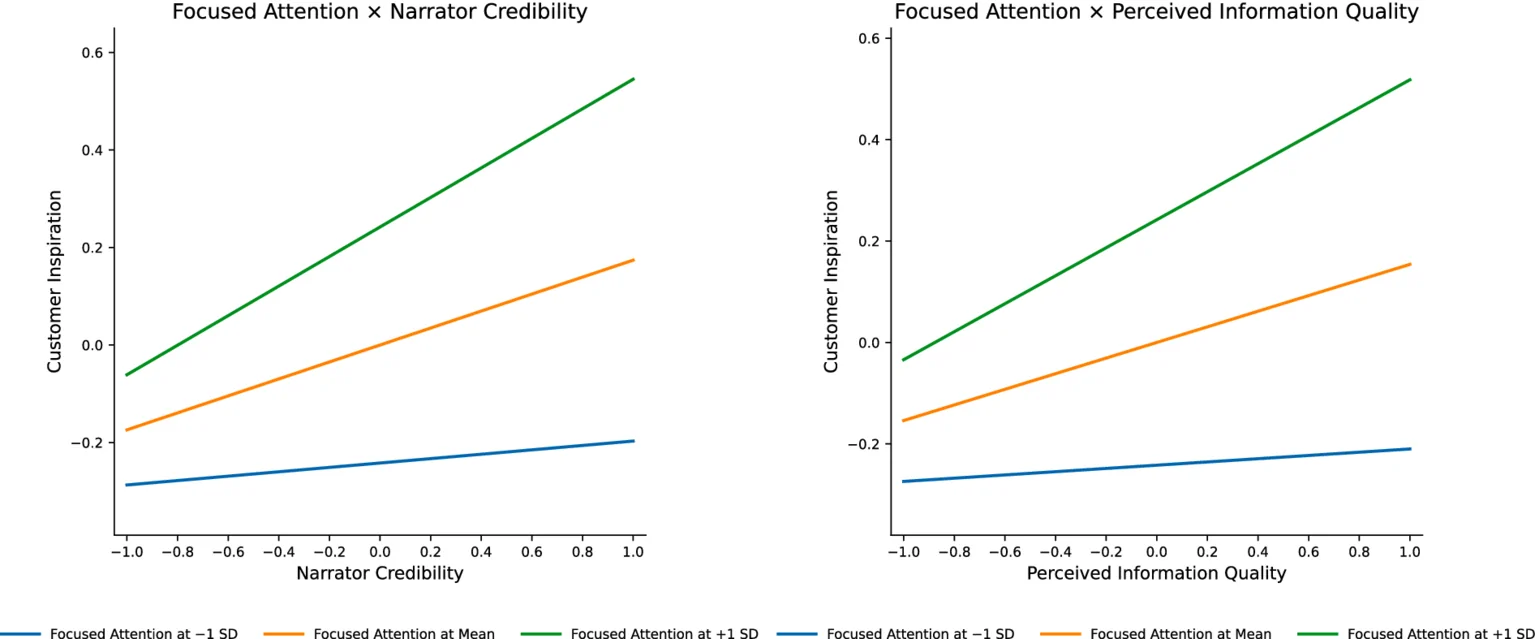
Moderating effects of focused attention.

Simple-slope analyses showed that the positive relationships of NC and PIQ with CI became steeper as FA increased from low (−1 SD) to mean and high (+1 SD) levels (Figure 5). At low FA (−1 SD), neither NC (*β* = 0.044, *p* = 0.451) nor PIQ (*β* = 0.032, *p* = 0.565) was significantly related to CI. At mean and high (+1 SD) levels of FA, both conditional effects were positive and significant, with the largest effects observed at high FA (NC: *β* = 0.303; PIQ: *β* = 0.276; both *p* < 0.001; Supplementary Table S6).

Mediation effects were tested using bootstrapping, with the results presented in Table 6. To determine the type of mediation, the direct effects of NNI and PNC on CI were additionally estimated in an extended mediation model. Both direct effects were significant: the direct effect of NNI on CI was *β* = 0.195, *t* = 4.740, *p* < 0.001, 95% CI [0.117, 0.279], and the direct effect of PNC on CI was *β* = 0.179, *t* = 4.142, *p* < 0.001, 95% CI [0.095, 0.265]. The indirect effects through NC (*β* = 0.026, *t* = 2.490, *p* = 0.013, 95% CI [0.007, 0.048]) and PIQ (*β* = 0.024, *t* = 1.973, *p* = 0.049, 95% CI [0.002, 0.050]) were also significant, supporting H2c and H3c. Because the corresponding direct and indirect effects were positive and significant, both pathways indicated complementary mediation. The sequential indirect effect of NNI on eWOMI through NC and CI was significant (*β* = 0.009, *t* = 2.271, *p* = 0.023, 95% CI [0.003, 0.019]). In contrast, the sequential indirect effect of PNC on eWOMI through PIQ and CI did not meet the predefined significance criterion (*β* = 0.009, *t* = 1.886, *p* = 0.059, 95% CI [0.001, 0.018]).

**Table 6:** Direct and indirect effects in the extended mediation model.

Hypothesis	Effect type	Path	*β*	*t*-value	*p*-value	95% CI	Result
–	Direct	NNI → CI	0.195	4.74	<0.001	[0.117, 0.279]	Significant
H2c	Indirect	NNI → NC → CI	0.026	2.49	0.013	[0.007, 0.048]	Complementary mediation
–	Direct	PNC → CI	0.179	4.142	<0.001	[0.095, 0.265]	Significant
H3c	Indirect	PNC → PIQ → CI	0.024	1.973	0.049	[0.002, 0.050]	Complementary mediation
–	Sequential indirect	NNI → NC → CI → eWOMI	0.009	2.271	0.023	[0.003, 0.019]	Significant
–	Sequential indirect	PNC → PIQ → CI → eWOMI	0.009	1.886	0.059	[0.001, 0.018]	Not significant

To further evaluate the model’s out-of-sample predictive ability, PLSpredict analysis was conducted. All *Q*^2^_predict values exceeded zero, indicating predictive relevance. However, the PLS-SEM model did not consistently produce lower prediction errors than the linear model benchmark across indicators, suggesting that the model demonstrated predictive capability, but its predictive advantage was limited (Supplementary Table S5).

### 4.4 Multigroup analysis

MICOM results based on 5,000 permutations established configural and compositional invariance for all constructs; however, equality of composite means and variances was not achieved for all constructs. Therefore, partial measurement invariance was established, supporting subsequent multigroup comparisons (Supplementary Table S3).

To determine whether the key structural relationships differed by narrator type, between-group differences were evaluated using permutation tests of the path-coefficient differences (Δ*β*), rather than differences in within-group significance levels. The relationship between NNI and NC differed significantly between conditions (human: *β* = 0.349; digital human: *β* = 0.114; Δ*β* = 0.235, permutation *p* = 0.001), supporting H6a. Similarly, the relationship between NC and CI differed significantly between conditions (human: *β* = 0.302; digital human: *β* = 0.062; Δ*β* = 0.240, permutation *p* = 0.001), supporting H6b. In contrast, the between-group differences in the relationships between PNC and PIQ (Δ*β* = 0.131, permutation *p* = 0.093) and between PIQ and CI (Δ*β* = 0.074, permutation *p* = 0.399) were not significant; therefore, H6c and H6d were not supported (Table 7).

**Table 7:** Multigroup analysis between human narrator and digital human narrator conditions.

Hypothesis	Path	Human *β*	Digital human *β*	Δ*β*	Permutation *p*-value	Result
H6a	NNI → NC	0.349	0.114	0.235	0.001	Supported
H6b	NC → CI	0.302	0.062	0.240	0.001	Supported
H6c	PNC → PIQ	0.315	0.184	0.131	0.093	Not supported
H6d	PIQ → CI	0.177	0.103	0.074	0.399	Not supported

Additionally, to further examine the specificity of the hypothesized paths, an exploratory extension analysis was conducted using the full sample. The complete methods and results are reported in Supplementary Section S4.

## 5 Discussion

### 5.1 Discussion of key findings

Participants evaluated the human narrator more favorably than the digital human narrator in terms of both nonverbal immediacy and narrator clarity. These group differences can be understood within the proposed dual-route psychological evaluation framework. Viewers formed evaluations of the narrator’s nonverbal presentation and verbal delivery based on facial expressions, bodily movements, gaze, vocal prosody, delivery fluency, and audiovisual coordination. Although the two videos were closely matched in script, duration, subtitles, background footage, and narrative structure, the human narrator’s bodily performance, vocal variation, and audiovisual coordination may have been perceived as more natural, coherent, and clear. This interpretation aligns with evidence that human-presented explainer videos receive more favorable user-experience evaluations than content-matched AI-avatar videos, possibly because human presenters provide more natural vocal and nonverbal cues (Netland et al., 2025; Reichert et al., 2026). Similarly, human anchors have been found to elicit stronger policy-adoption intentions than digital anchors, partly due to differences in perceived psychological distance (Chen et al., 2025). These findings suggest that viewers respond not only to the category of the communication source but also to how they perceive and interpret specific presentation cues. However, because the present study compared two complete video conditions without independently manipulating narrator identity, voice, facial expressions, or bodily movements, the results indicate only that the human narrator’s overall presentation received more favorable evaluations under the specific stimulus and production conditions used in this study. They do not isolate the contribution of any individual cue or establish that this advantage applies to all ICH short-form video contexts.

The multigroup analysis revealed that narrator-type differences were primarily concentrated in the source-evaluation route rather than the information-evaluation route. One possible explanation is that source evaluation relies more on interpersonal cues such as naturalness, sincerity, and social presence, which may favor human narrators. Prior research also suggests that virtual influencers may be perceived as less culturally grounded, while sincerity, expertise, and attractiveness can enhance credibility-related evaluations and inspire customers. In contrast, the information-evaluation route may depend more on content clarity and quality. Since both conditions used the same script, narrative structure, and background information, this route was likely less sensitive to narrator type.

Beyond the between-group pattern, the dual-route model suggests that customer inspiration can arise from evaluations of either the communicator or the conveyed information. This distinction extends prior short-form video research, which separates source-related cues from information-quality cues (Yang et al., 2025), by demonstrating how both evaluation targets converge to inspire customers in ICH communication. The relatively small indirect effects suggest that narrator credibility and perceived information quality represent partial rather than dominant mechanisms linking narrator cues to customer inspiration. Given the large sample size, the significant relationships should be interpreted alongside their generally small effect sizes.

Customer inspiration may further serve as a motivational bridge between favorable evaluations and eWOMI. Evaluating a narrator as credible or the information as high quality does not, by itself, imply a willingness to share; rather, these evaluations may encourage dissemination when they generate interest and a desire for further engagement. This interpretation aligns with evidence linking customer inspiration to eWOM (Ibrahim et al., 2025).

Focused attention appears to condition whether favorable source and information evaluations develop into inspiration. Although the interaction effects were small, the simple-slope results suggest that focused attention serves as a modest but meaningful boundary condition, with stronger evaluation–inspiration associations at higher attention levels. Greater attentional engagement may allow viewers to process and integrate narrator- and content-related information more deeply, thereby strengthening their motivational responses. This interpretation is consistent with evidence that rapid context switching in short-form video environments can impair information processing and behavioral intentions (Chiossi et al., 2023). However, because focused attention was measured subjectively, this interpretation should be treated cautiously and validated using objective measures such as eye tracking, fixation duration, or cognitive load.

### 5.2 Implications

The primary theoretical contribution of this study is to extend source credibility theory to AI-mediated cultural communication. Rather than treating human and digital human narrators as simple source categories, this study identifies narrator nonverbal immediacy as a perceptual cue linked to narrator credibility and subsequent customer inspiration. The study also extends customer inspiration research by demonstrating that inspiration in cultural heritage communication can arise from both source-related and information-related evaluations. By integrating narrator credibility and perceived information quality within a dual-route framework, the study elucidates how evaluations of both the communicator and the conveyed information contribute to customer inspiration and subsequent electronic word-of-mouth intention. Moreover, the stronger human narrator advantage in the source-evaluation route suggests that AI-mediated communication may depend on whether viewers focus on evaluating the communicator or the conveyed information. Finally, the findings on focused attention further indicate that the translation of favorable evaluations into inspiration partly depends on viewers’ attentional engagement.

The data-supported practical implications of this study suggest that narrator selection should align with communication objectives. Human narrators may be especially valuable when the goal is to enhance source-related evaluations, such as narrator credibility and customer inspiration. In contrast, because the information-evaluation route did not differ significantly between narrator conditions, perceived narrator clarity and information quality should be prioritized regardless of narrator type in knowledge-focused ICH communication. The findings on focused attention suggest that viewers’ attentional engagement may be important for translating favorable evaluations into inspiration.

Beyond these data-supported implications, broader design recommendations can be drawn from prior research in multimedia communication. Strategies such as coordinating speech, lip movements, gaze, and gestures, as well as using highlighted subtitles, close-up visuals, segmented knowledge units, and contextually relevant prompts, may enhance comprehension and engagement. However, these design strategies were not directly tested in the present study and should therefore be considered literature-based recommendations that require further empirical validation. Regardless of narrator type, accurate, representative, and comprehensible cultural information remains important for ICH communication.

### 5.3 Limitations and future research

This study has several limitations. First, participants were recruited through convenience sampling, and the sample was concentrated in the 25–38-year age range, which may limit the generalizability of the findings. Future research should include more diverse age groups, cultural backgrounds, and platforms. Second, the experiment focused on a single ICH context (Hakka tulou) and used only one human narrator and one digital human narrator; thus, characteristics such as appearance, voice, and presentation style may have influenced the results. Accordingly, the findings should be generalized cautiously beyond the specific Hakka tulou context, narrator pair, and convenience sample examined in this study. Future studies should employ multiple human and digital human narrators, examine different types of ICH, and use factorial designs to independently manipulate cues such as voice, movement, and gaze.

Third, the study relied mainly on post-viewing self-report measures, with mediators and outcomes assessed within the same survey. Because only narrator type was experimentally manipulated, the relationships among the post-exposure self-report constructs should be interpreted as associations rather than causal relationships. This may increase common method bias and limit conclusions about temporal ordering. Subjective focused attention may also not fully reflect actual attention, and eWOMI may not translate into actual sharing behavior. Future studies could use temporally separated measurements, eye tracking, behavioral logs, and actual sharing behaviors.

Although the model identified significant relationships, the relatively modest *R*^2^ values indicate that narrator-related cues explain only part of viewers’ responses. Likewise, the small but significant indirect effects suggest that the proposed mediating mechanisms represent partial rather than dominant pathways to customer inspiration. Future research could incorporate additional individual and contextual factors to improve the explanatory power of the model.

Future research should also examine unmeasured perceptual mechanisms, including perceived realism, attractiveness, anthropomorphism, social presence, and uncanny-valley responses, which may account for additional variation in how viewers respond to human and digital human narrators, particularly in source evaluation.

## 6 Conclusion

This study employed explainer-style ICH short-form videos featuring Hakka tulou to compare human and digital human narrators and examine relationships among narrator presentation cues, viewer evaluations, and electronic word-of-mouth intention. Within the stimuli examined, participants reported higher narrator nonverbal immediacy and perceived narrator clarity in the human narrator condition. Narrator nonverbal immediacy was associated with customer inspiration through narrator credibility, whereas perceived narrator clarity was associated with customer inspiration through perceived information quality; customer inspiration was positively associated with electronic word-of-mouth intention. The human narrator advantage was concentrated in the source-evaluation pathway, with stronger relationships between narrator nonverbal immediacy and narrator credibility and between narrator credibility and customer inspiration, while the information-evaluation relationships did not differ significantly between narrator conditions. Focused attention was associated with stronger evaluation–inspiration relationships. Overall, these findings suggest that, within the specific stimuli and sample examined, differences between human and digital human narrators were more evident in source evaluation than in information evaluation and may inform the design of ICH short-form videos.

## Data Availability

The de-identified datasets generated and analyzed during this study are available from the corresponding author upon reasonable request.
