# Supplementary material for: Human and digital human narrators in intangible cultural heritage short videos: dual psychological evaluation pathways through customer inspiration to electronic word-of-mouth intention
Source: Front Psychol. 2026 Oct 26;17:1960625. doi: 10.3389/fpsyg.2026.1960625 (PMC13745517; doi:10.3389/fpsyg.2026.1960625)
Supplement: Supplementary file 1 [file Supplementary_file_1.DOCX]

***Supplementary Material***

**Supplementary Tables**

**Table S1.** Measurement Items and Single-Item Background Measures

The measurement model included five items for NNI, five items for PNC, five items for NC, five items for PIQ, six items for CI, five items for eWOMI, and four items for FA.

| **Construct/Item** | **Measurement item** | **Source** | **Adaptation/**  **Modification** |
| --- | --- | --- | --- |
| NNI | The narrator in the video used hand gestures to support the explanation. | Richmond et al. (2003) | Contextualized the original interpersonal communication item to video-based cultural narration. |
|  | The narrator’s tone and rhythm varied naturally rather than sounding monotonous or mechanical. | Richmond et al. (2003) | Adapted vocal-expression wording to fit narrator performance in short-form videos. |
|  | The narrator naturally looked toward the audience or the camera. | Richmond et al. (2003) | Replaced face-to-face interaction context with camera-based video viewing context. |
|  | The narrator appeared vivid and energetic while explaining the content. | Richmond et al. (2003) | Modified wording to describe narrator presentation during ICH video explanation. |
|  | The narrator showed natural and friendly facial expressions while explaining the content. | Richmond et al. (2003) | Adapted nonverbal behavior description to digital/video narration context. |
| PNC | The narrator clearly explained the important concepts related to intangible cultural heritage. | Chesebro and McCroskey (1998) | Replaced “teacher/course concepts” with “narrator/ICH concepts.” |
|  | Overall, I could understand the narrator’s explanation in the video. | Chesebro and McCroskey (1998) | Changed classroom learning context to short-video viewing context. |
|  | The narrator’s explanation was straightforward. | Chesebro and McCroskey (1998) | Retained the original clarity dimension and adapted the subject from teacher to narrator. |
|  | The narrator used clear and relevant explanations to help me understand the content. | Chesebro and McCroskey (1998) | Adapted instructional examples to cultural-content explanation. |
|  | The narrator expressed the key points clearly. | Chesebro and McCroskey (1998) | Directly adopted with minor contextual clarification. |
| NC | The narrator was trustworthy. | Ohanian (1990) | Added ICH explanation context while retaining credibility meaning. |
|  | The narrator was sincere when explaining intangible cultural heritage content. | Ohanian (1990) | Added cultural explanation context. |
|  | The narrator was professional in explaining intangible cultural heritage content. | Ohanian (1990) | Expanded expertise description to match ICH knowledge requirements. |
|  | The narrator had rich knowledge of intangible cultural heritage techniques, historical background, and cultural meaning. | Ohanian (1990) | Added ICH explanation context. |
|  | The narrator was qualified to explain intangible cultural heritage content. | Ohanian (1990) | Replaced general information evaluation context with ICH information context. |
| PIQ | The intangible cultural heritage content in the video was relevant to my need to understand this heritage item. | Cheung et al. (2008) | Adapted information evaluation to cultural video content. |
|  | The intangible cultural heritage content in the video was appropriate and applicable. | Cheung et al. (2008) | Added ICH content context while preserving information-quality meaning. |
|  | The intangible cultural heritage content in the video was accurate and reliable. | Cheung et al. (2008) | Expanded completeness dimension to reflect ICH learning needs. |
|  | The intangible cultural heritage content in the video sufficiently met my need to understand the related techniques, historical background, and cultural meaning. | Cheung et al. (2008) | Adapted inspiration measurement from consumption context to cultural-learning context. |
|  | The intangible cultural heritage content in the video had sufficient knowledge breadth and narrative depth. | Cheung et al. (2008) | Replaced product/service inspiration context with cultural-content inspiration. |
| CI | After watching this intangible cultural heritage short video, my imagination was stimulated. | Böttger et al. (2017) | Adapted cognitive inspiration to cultural heritage understanding. |
|  | After watching this intangible cultural heritage short video, I was intrigued by the new ideas presented in it. | Böttger et al. (2017) | Replaced purchase-oriented motivation with cultural exploration motivation. |
|  | After watching this intangible cultural heritage short video, my understanding of the related heritage culture was broadened. | Böttger et al. (2017) | Adapted behavioral motivation toward heritage engagement. |
|  | After watching this intangible cultural heritage short video, I was inspired to learn more about related heritage content. | Böttger et al. (2017) | Replaced consumption behavior with cultural exploration behavior. |
|  | After watching this intangible cultural heritage short video, my interest in related heritage content increased. | Böttger et al. (2017) | Contextualized the original interpersonal communication item to video-based cultural narration. |
|  | After watching this intangible cultural heritage short video, I was motivated to explore more related heritage content. | Böttger et al. (2017) | Adapted vocal-expression wording to fit narrator performance in short-form videos. |
| eWOMI | I would recommend this intangible cultural heritage short video or the heritage content introduced in it to others. | Goyette et al. (2010) | Replaced face-to-face interaction context with camera-based video viewing context. |
|  | I would say positive things about this intangible cultural heritage short video or the heritage content introduced in it. | Goyette et al. (2010) | Modified wording to describe narrator presentation during ICH video explanation. |
|  | I would share this intangible cultural heritage short video with people who usually pay little attention to intangible cultural heritage, traditional culture, or museum-related content. | Goyette et al. (2010) | Adapted nonverbal behavior description to digital/video narration context. |
|  | I would share this intangible cultural heritage short video on social media, group chats, or Moments so that people from broader interest circles could see it. | Goyette et al. (2010) | Replaced “teacher/course concepts” with “narrator/ICH concepts.” |
|  | I would talk with others about the cultural highlights, cultural value, or interesting aspects presented in this intangible cultural heritage short video. | Goyette et al. (2010) | Changed classroom learning context to short-video viewing context. |
| FA | While watching this intangible cultural heritage short video, I was fully immersed in the content. | O’Brien et al. (2018) | Retained the original clarity dimension and adapted the subject from teacher to narrator. |
|  | While watching this video, time seemed to pass without my noticing. | O’Brien et al. (2018) | Adapted instructional examples to cultural-content explanation. |
|  | While watching this video, I was fully absorbed in it. | O’Brien et al. (2018) | Directly adopted with minor contextual clarification. |
|  | While watching this video, I blocked out the surrounding environment and focused on the video. | O’Brien et al. (2018) | Added ICH explanation context while retaining credibility meaning. |
| Single Viewing Time (SVT) | What is your average viewing duration when you watch intangible cultural heritage, traditional culture, or museum-related short videos? 1 = Less than 1 min; 2 = 1–2 min; 3 = 2–3 min; 4 = 3–5 min; 5 = More than 5 min. | Single-item self-report | Added cultural explanation context. |
| Cumulative Exposure Frequency (CEF) | How often have you watched intangible cultural heritage, traditional culture, or museum-related short videos in the past month? 1 = Never; 2 = 1–2 times; 3 = 3–5 times; 4 = 6–10 times; 5 = More than 10 times. | Single-item self-report | Expanded expertise description to match ICH knowledge requirements. |

**Table S2.** Cross-loadings of Measurement Items

| **Item** | **PIQ** | **eWOMI** | **FA** | **NC** | **PNC** | **NNI** | **CI** |
| --- | --- | --- | --- | --- | --- | --- | --- |
| **eWOMI1** | 0.117 | **0.741** | 0.226 | 0.185 | 0.133 | 0.181 | 0.22 |
| **eWOMI2** | 0.169 | **0.739** | 0.27 | 0.242 | 0.228 | 0.23 | 0.267 |
| **eWOMI3** | 0.233 | **0.756** | 0.262 | 0.249 | 0.194 | 0.232 | 0.274 |
| **eWOMI4** | 0.202 | **0.741** | 0.28 | 0.248 | 0.214 | 0.215 | 0.288 |
| **eWOMI5** | 0.205 | **0.712** | 0.277 | 0.213 | 0.179 | 0.272 | 0.262 |
| **FA1** | 0.161 | 0.283 | **0.731** | 0.151 | 0.08 | 0.14 | 0.159 |
| **FA2** | 0.214 | 0.255 | **0.78** | 0.199 | 0.191 | 0.214 | 0.24 |
| **FA3** | 0.212 | 0.293 | **0.795** | 0.174 | 0.171 | 0.211 | 0.236 |
| **FA4** | 0.266 | 0.271 | **0.747** | 0.223 | 0.188 | 0.206 | 0.255 |
| **NNI1** | 0.131 | 0.209 | 0.139 | 0.144 | 0.169 | **0.749** | 0.171 |
| **NNI2** | 0.292 | 0.228 | 0.225 | 0.207 | 0.213 | **0.765** | 0.275 |
| **NNI3** | 0.193 | 0.209 | 0.193 | 0.158 | 0.245 | **0.732** | 0.329 |
| **NNI4** | 0.15 | 0.222 | 0.185 | 0.214 | 0.27 | **0.773** | 0.275 |
| **NNI5** | 0.182 | 0.276 | 0.209 | 0.19 | 0.228 | **0.715** | 0.271 |
| **PNC1** | 0.171 | 0.139 | 0.161 | 0.207 | **0.753** | 0.147 | 0.188 |
| **PNC2** | 0.21 | 0.218 | 0.208 | 0.222 | **0.767** | 0.289 | 0.257 |
| **PNC3** | 0.208 | 0.174 | 0.135 | 0.232 | **0.773** | 0.247 | 0.241 |
| **PNC4** | 0.209 | 0.24 | 0.165 | 0.283 | **0.769** | 0.249 | 0.303 |
| **PNC5** | 0.197 | 0.202 | 0.146 | 0.265 | **0.716** | 0.204 | 0.326 |
| **NC1** | 0.12 | 0.184 | 0.162 | **0.756** | 0.189 | 0.163 | 0.168 |
| **NC2** | 0.216 | 0.275 | 0.219 | **0.739** | 0.272 | 0.226 | 0.186 |
| **NC3** | 0.173 | 0.233 | 0.178 | **0.766** | 0.225 | 0.163 | 0.25 |
| **NC4** | 0.187 | 0.168 | 0.184 | **0.734** | 0.244 | 0.201 | 0.212 |
| **NC5** | 0.185 | 0.28 | 0.172 | 0.699 | 0.247 | 0.164 | 0.236 |
| **PIQ1** | **0.751** | 0.142 | 0.184 | 0.116 | 0.169 | 0.133 | 0.156 |
| **PIQ2** | **0.756** | 0.199 | 0.249 | 0.174 | 0.205 | 0.172 | 0.207 |
| **PIQ3** | **0.766** | 0.227 | 0.201 | 0.19 | 0.205 | 0.242 | 0.234 |
| **PIQ4** | **0.759** | 0.165 | 0.193 | 0.205 | 0.174 | 0.189 | 0.22 |
| **PIQ5** | **0.665** | 0.199 | 0.228 | 0.199 | 0.219 | 0.206 | 0.156 |
| **CI1** | 0.16 | 0.163 | 0.133 | 0.144 | 0.22 | 0.179 | **0.7** |
| **CI2** | 0.194 | 0.317 | 0.247 | 0.266 | 0.324 | 0.282 | **0.75** |
| **CI3** | 0.192 | 0.233 | 0.243 | 0.239 | 0.283 | 0.309 | **0.772** |
| **CI4** | 0.203 | 0.274 | 0.213 | 0.195 | 0.252 | 0.267 | **0.728** |
| **CI5** | 0.148 | 0.268 | 0.234 | 0.208 | 0.256 | 0.243 | **0.725** |
| **CI6** | 0.256 | 0.265 | 0.201 | 0.17 | 0.173 | 0.247 | **0.677** |

**Table S3.** MICOM Measurement Invariance Assessment

| **Construct** | **Step 2 p-Value** | **Compositional Invariance** | **Step 3a p-Value** | **Equal Means** | **Step 3b p-Value** | **Equal Variances** |
| --- | --- | --- | --- | --- | --- | --- |
| PIQ | 0.186 | Yes | <0.001 | No | 0.290 | Yes |
| eWOMI | 0.296 | Yes | <0.001 | No | 0.926 | Yes |
| FA | 0.327 | Yes | <0.001 | No | 0.788 | Yes |
| NC | 0.071 | Yes | <0.001 | No | <0.001 | No |
| PNC | 0.223 | Yes | <0.001 | No | 0.020 | No |
| NNI | 0.278 | Yes | <0.001 | No | 0.188 | Yes |
| CI | 0.293 | Yes | <0.001 | No | 0.583 | Yes |

**Table S4.** HTMT Estimates and 95% Bootstrap Confidence Intervals

| **Construct Pair** | **Original Sample (O)** | **Sample Mean (M)** | **2.5%** | **97.50%** |
| --- | --- | --- | --- | --- |
| **eWOMI ↔ PIQ** | 0.312 | 0.314 | 0.217 | 0.41 |
| **FA ↔ PIQ** | 0.357 | 0.357 | 0.266 | 0.45 |
| **FA ↔ eWOMI** | 0.46 | 0.46 | 0.368 | 0.551 |
| **NC ↔ PIQ** | 0.297 | 0.298 | 0.206 | 0.392 |
| **NC ↔ eWOMI** | 0.385 | 0.385 | 0.293 | 0.477 |
| **NC ↔ FA** | 0.311 | 0.312 | 0.22 | 0.409 |
| **PNC ↔ PIQ** | 0.326 | 0.327 | 0.233 | 0.421 |
| **PNC ↔ eWOMI** | 0.317 | 0.318 | 0.218 | 0.417 |
| **PNC ↔ FA** | 0.261 | 0.265 | 0.171 | 0.358 |
| **PNC ↔ NC** | 0.395 | 0.395 | 0.308 | 0.485 |
| **NNI ↔ PIQ** | 0.313 | 0.315 | 0.228 | 0.406 |
| **NNI ↔ eWOMI** | 0.381 | 0.383 | 0.299 | 0.469 |
| **NNI ↔ FA** | 0.317 | 0.319 | 0.229 | 0.41 |
| **NNI ↔ NC** | 0.304 | 0.307 | 0.215 | 0.399 |
| **NNI ↔ PNC** | 0.367 | 0.369 | 0.277 | 0.46 |
| **CI ↔ PIQ** | 0.323 | 0.323 | 0.231 | 0.416 |
| **CI ↔ eWOMI** | 0.429 | 0.429 | 0.342 | 0.513 |
| **CI ↔ FA** | 0.358 | 0.359 | 0.272 | 0.442 |
| **CI ↔ NC** | 0.344 | 0.344 | 0.26 | 0.429 |
| **CI ↔ PNC** | 0.42 | 0.42 | 0.336 | 0.504 |
| **CI ↔ NNI** | 0.426 | 0.428 | 0.346 | 0.513 |

**Table S5.** PLSpredict Results

| **Indicator** | **Q²_predict** | **PLS-SEM_RMSE** | **PLS-SEM_MAE** | **LM_RMSE** | **LM_MAE** |
| --- | --- | --- | --- | --- | --- |
| **Q7_Row1** | 0.024 | 1.147 | 0.848 | 1.152 | 0.865 |
| **Q7_Row2** | 0.039 | 0.956 | 0.782 | 0.953 | 0.763 |
| **Q7_Row3** | 0.038 | 0.935 | 0.762 | 0.921 | 0.723 |
| **Q7_Row4** | 0.026 | 0.989 | 0.801 | 0.965 | 0.753 |
| **Q7_Row5** | 0.043 | 0.905 | 0.737 | 0.886 | 0.688 |
| **Q10_Row1** | 0.033 | 1.148 | 0.826 | 1.144 | 0.879 |
| **Q10_Row2** | 0.042 | 0.952 | 0.79 | 0.936 | 0.722 |
| **Q10_Row3** | 0.041 | 0.938 | 0.784 | 0.915 | 0.718 |
| **Q10_Row4** | 0.042 | 0.937 | 0.78 | 0.905 | 0.716 |
| **Q10_Row5** | 0.041 | 0.982 | 0.815 | 0.96 | 0.749 |
| **Q6_Row1** | 0.022 | 1.143 | 0.841 | 1.138 | 0.862 |
| **Q6_Row2** | 0.046 | 0.914 | 0.735 | 0.899 | 0.693 |
| **Q6_Row3** | 0.023 | 0.947 | 0.756 | 0.939 | 0.727 |
| **Q6_Row4** | 0.036 | 0.943 | 0.769 | 0.935 | 0.735 |
| **Q6_Row5** | 0.023 | 0.947 | 0.767 | 0.94 | 0.73 |
| **Q9_Row1** | 0.036 | 1.162 | 0.86 | 1.153 | 0.881 |
| **Q9_Row2** | 0.091 | 0.946 | 0.765 | 0.915 | 0.697 |
| **Q9_Row3** | 0.089 | 0.923 | 0.753 | 0.886 | 0.696 |
| **Q9_Row4** | 0.071 | 0.934 | 0.757 | 0.926 | 0.719 |
| **Q9_Row5** | 0.081 | 0.941 | 0.771 | 0.936 | 0.73 |
| **Q9_Row6** | 0.058 | 0.943 | 0.769 | 0.938 | 0.744 |

**Table S6.** Conditional Effects at Low, Mean, and High Levels of Focused Attention

| **Predictor → CI** | **FA level** | **Conditional effect** | **t** | **p** | **95% CI** |
| --- | --- | --- | --- | --- | --- |
| PIQ → CI | Low (−1 SD) | 0.032 | 0.576 | 0.565 | [−0.076, 0.141] |
| PIQ → CI | Mean | 0.154 | 3.644 | <.001 | [0.072, 0.239] |
| PIQ → CI | High (+1 SD) | 0.276 | 4.898 | <.001 | [0.168, 0.389] |
| NC → CI | Low (−1 SD) | 0.044 | 0.754 | 0.451 | [−0.064, 0.168] |
| NC → CI | Mean | 0.174 | 4.67 | <.001 | [0.104, 0.248] |
| NC → CI | High (+1 SD) | 0.303 | 6.465 | <.001 | [0.209, 0.393] |

**S4. Exploratory Pathway-Specificity Analysis**

To examine whether the two types of presentation cues were related only to their hypothesized proximal evaluation targets, an exploratory extension analysis was conducted using the full sample (N = 601). Building on the main model, two cross-paths—NNI → PIQ and PNC → NC—were added simultaneously, and the model was estimated using the same PLS-SEM procedure and 5,000 bootstrap resamples as in the main analysis. Because this was a post hoc exploratory test, the results were used only to assess the specificity of the hypothesized paths and not to re-evaluate the study hypotheses. The inner VIF values for the predictors of NC and PIQ were both 1.100, indicating no substantial multicollinearity.

Results showed that both hypothesized paths remained significant: NNI was significantly associated with NC (β = 0.167, p < 0.001), and PNC was significantly associated with PIQ (β = 0.202, p < 0.001). The two cross-paths were also significant: NNI was associated with PIQ (β = 0.208, p < 0.001), and PNC was associated with NC (β = 0.274, p < 0.001; see Figure S1 and Table S4). All four specific indirect effects through NC or PIQ to CI were significant (Table S5). Thus, the two evaluation constructs can be distinguished as different proximal targets, but the presentation cues and evaluation targets are not in a one-to-one or mutually exclusive relationship. Because the path coefficients in the extended model are conditional effects estimated while controlling for the other presentation cue, differences from the coefficients in the main model are expected.

**Figure S1.** Results of the Exploratory Extended Model

**Table S4.** Path Coefficients of the Exploratory Extended Model

| **Path** | **β** | **t-value** | **p-value** | **95% CI** | **f²** |
| --- | --- | --- | --- | --- | --- |
| **NNI → NC** | 0.167 | 4.084 | <.001 | [.088, .249] | 0.029 |
| **PNC → PIQ** | 0.202 | 4.700 | <.001 | [.119, .288] | 0.042 |
| **NNI → PIQ** | 0.208 | 5.204 | <.001 | [.132, .288] | 0.044 |
| **PNC → NC** | 0.274 | 6.701 | <.001 | [.196, .356] | 0.078 |
| **NC → CI** | 0.173 | 4.695 | <.001 | [.102, .246] | 0.032 |
| **PIQ → CI** | 0.154 | 3.635 | <.001 | [.071, .237] | 0.025 |
| **CI → eWOMI** | 0.358 | 11.021 | <.001 | [.298, .425] | 0.147 |

**Table S5.** Specific Indirect Effects

| **Specific Indirect Path** | **β** | **t-value** | **p-value** | **95% CI** |
| --- | --- | --- | --- | --- |
| **NNI → NC → CI** | 0.029 | 2.849 | 0.004 | [.012, .052] |
| **PNC → PIQ → CI** | 0.031 | 2.620 | 0.009 | [.011, .058] |
| **NNI → PIQ → CI** | 0.032 | 2.878 | 0.004 | [.013, .056] |
| **PNC → NC → CI** | 0.047 | 3.851 | <.001 | [.026, .074] |
